# (*E*)-3-Isopropyl-1-methyl-2,6-di­phenyl­piperidin-4-one *O*-nicotinoyl oxime

**DOI:** 10.1107/S1600536814007363

**Published:** 2014-04-12

**Authors:** T. Vinuchakkaravarthy, R. Sivakumar, T. Srinivasan, V. Thanikachalam, D. Velmurugan

**Affiliations:** a Centre of Advanced Study in Crystallography and Biophysics, University of Madras, Maraimalai Campus (Guindy Campus), Chennai 600 025, India; b Department of Chemistry, Annamalai University, Annamalai Nagar, Chidambaram 608 002, India

## Abstract

In the title compound, C_27_H_29_N_3_O_2_, the piperidine ring exists in a chair conformation with an equatorial orientation of the phenyl and methyl substituents. The C—C=N bond angles are significantly different [119.1 (2) and 127.2 (2)°]. The phenyl rings are inclined to one another by 44.90 (14)°, and by 80.85 (13) and 79.62 (12)° to the mean plane of the piperidine ring. The terminal pyridine ring is inclined to the piperidine ring mean plane by 74.79 (15)°. In the crystal, mol­ecules are linked by C—H⋯π inter­actions, forming a three-dimensional network.

## Related literature   

For the synthesis and biological activity of piperidin-4-one derivatives, see, for example: Parthiban *et al.* (2009 ▶); Narayanan *et al.* (2012 ▶). For the crystal structures of very similar compounds, see: Vinuchakkaravarthy *et al.* (2013*a* ▶,*b* ▶). For ring puckering parameters, see: Cremer & Pople (1975 ▶); Nardelli (1983 ▶).
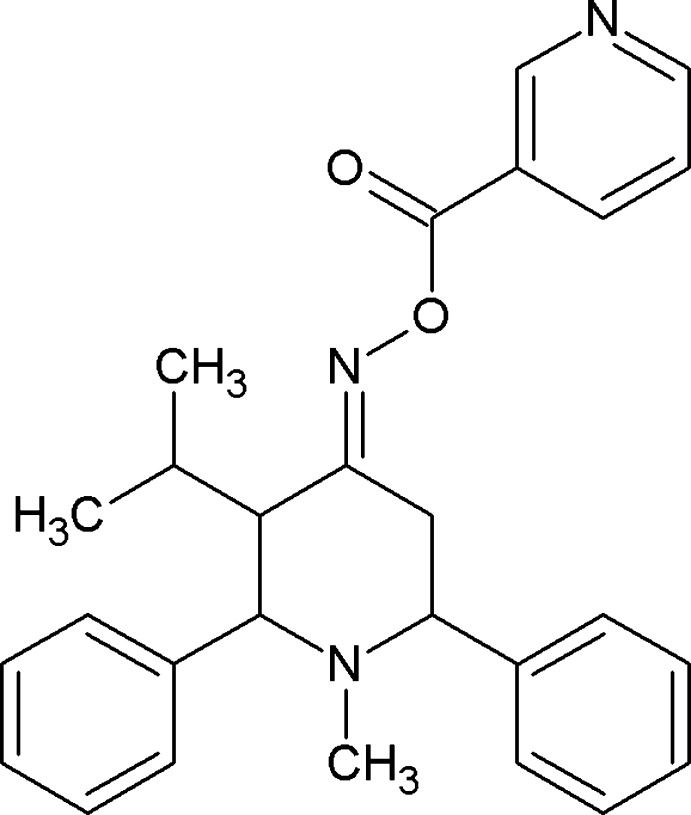


## Experimental   

### 

#### Crystal data  

C_27_H_29_N_3_O_2_
*M* *_r_* = 427.53
Orthorhombic, 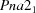

*a* = 12.7717 (6) Å
*b* = 16.2765 (7) Å
*c* = 11.4109 (9) Å
*V* = 2372.1 (2) Å^3^
*Z* = 4
Mo *K*α radiation
μ = 0.08 mm^−1^
*T* = 293 K
0.20 × 0.20 × 0.20 mm

#### Data collection  

Bruker SMART APEXII CCD diffractometer
Absorption correction: multi-scan (*SADABS*; Bruker, 2008 ▶) *T*_min_ = 0.491, *T*_max_ = 0.746
11654 measured reflections
5117 independent reflections
2975 reflections with *I* > 2σ(*I*)
*R*_int_ = 0.045

#### Refinement  

*R*[*F* ^2^ > 2σ(*F* ^2^)] = 0.047
*wR*(*F* ^2^) = 0.132
*S* = 1.03
5117 reflections
292 parameters
1 restraint
H-atom parameters constrained
Δρ_max_ = 0.15 e Å^−3^
Δρ_min_ = −0.14 e Å^−3^

### 

Data collection: *APEX2* (Bruker, 2008 ▶); cell refinement: *SAINT* (Bruker, 2008 ▶); data reduction: *SAINT*; program(s) used to solve structure: *SHELXS97* (Sheldrick, 2008 ▶); program(s) used to refine structure: *SHELXL97* (Sheldrick, 2008 ▶); molecular graphics: *ORTEP-3 for Windows* (Farrugia, 2012 ▶); software used to prepare material for publication: *SHELXL97* and *PLATON* (Spek, 2009 ▶).

## Supplementary Material

Crystal structure: contains datablock(s) global, I. DOI: 10.1107/S1600536814007363/su2680sup1.cif

Structure factors: contains datablock(s) I. DOI: 10.1107/S1600536814007363/su2680Isup2.hkl

Supporting information file. DOI: 10.1107/S1600536814007363/su2680Isup3.cml

CCDC reference: 995062

Additional supporting information:  crystallographic information; 3D view; checkCIF report

## Figures and Tables

**Table 1:** Hydrogen-bond geometry (Å, °) *Cg*1 and *Cg*2 are the centroids of rings C6–C11 and C22–C27, respectively.

*D*—H⋯*A*	*D*—H	H⋯*A*	*D*⋯*A*	*D*—H⋯*A*
C24—H24⋯*Cg*1^i^	0.93	2.87	3.700 (4)	150
C13—H13*C*⋯*Cg*2^ii^	0.96	2.95	3.620 (3)	128

Symmetry codes: (i) 
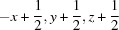
; (ii) 
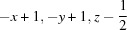
.

**Fig. 1.**
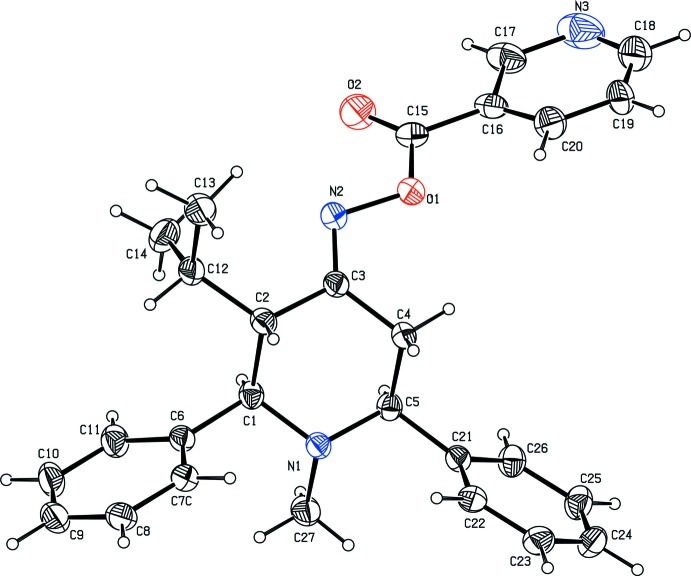
The molecular structure of the title molecule, with atom labelling. Displacement ellipsoids are drawn at the 30% probability level.

**Fig. 2.**
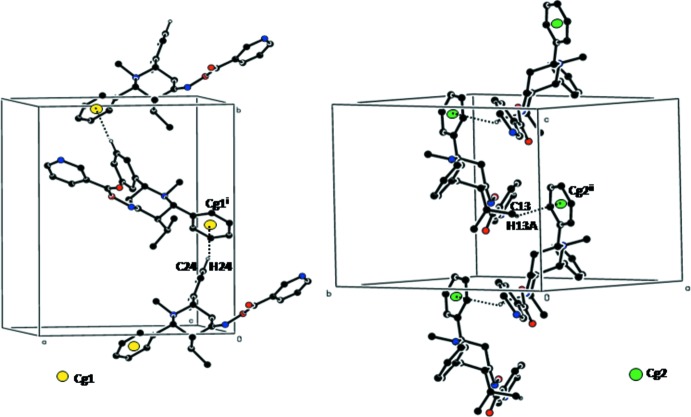
Two views of the crystal packing of the title compound, showing the C—H···π interactions (dashed lines; see Table 1 for details; symmetry codes: (i) 1/2 - x, 1/2 y + z; (ii) -x, -y, 1/2 - z)

## supplementary crystallographic information

### 1. Comment

The piperdin-4-one nucleus is an important class of pharmacophore due to its broad spectrum of biological actions ranging from antibacterial to anticancer (Parthiban *et al.*, 2009; Narayanan *et al.*, (2012). Hence, their synthesis and steriochemical analysis has gained much interest in the field of medicinal chemistry. Continuing our interest in such compounds (Vinuchakkaravarthy *et al.*, 2013*a*,*b*) we have synthesized the title compound and report herein on its crystal structure.

The molecular structure of the title molecule is shown in Fig. 1. The piperidine ring N1/C1-C5 adopts a chair conformation with the deviations of atoms N1 and C3 from the mean plane through atoms C1/C2/C4/C5 being -0.6131 (19) and 0.6448 (25) Å, respectively. The smallest displacement asymmetry parameters (Nardelli, 1983) q2 and q3 are 0.024 (2) and -0.552 (2) Å. The ring puckering parameters (Cremer & Pople, 1975) Q_T_ and phase angle θ are 0.553 (2) and 178.6 (2)°, respectively. Thus, all parameters support the chair conformation of piperidine ring.

The C—C═N bond angles are significantly different [119.1 (2) and 127.2 (2)°]. The phenyl rings (C6-C11 and C22-C27) are inclined to one another by and 44.90 (14)°, and by 80.85 (13) and 79.62 (12)°, respectively, to the mean plane of the piperidine ring (N1/C1-C5). The terminal pyridine ring (N3/C21/C16-C19) is inclined to the piperidine ring (N1/C1-C5) mean plane by 44.90 (14)°.

In the crystal, the molecules are linked by C-H···π interactions (Table 1and Fig. 2) forming a three-dimensional structure.

### 2. Experimental

The intermediate 3-ethyl-2,6-diphenylpiperidin-4-one (I) was synthesized by Mannich condensation using benzaldehyde (2 mol), ammonium acetate (1 mol) and ethyl methyl ketone (1 mol) in absolute ethanol and warmed for 30 min and stirred overnight at room temperature. The product obtained was treated with methyl iodide in the presence of potassium carbonate and refluxed to give (I). The oximation of (I) was carried out by adding hydroxylamine hydrochloride in the presence of sodium acetate in ethanol and the mixture was refluxed for 2h. The resulting oxime (0.5 g, 1.55 mmol) was stirred with dry pyridine (5 ml), then 3-methylbenzoic acid (0.21 g, 1.7 mmol) was added followed by drop wise addition of phosphorus oxychloride (0.21 mL, 2.3 mmol) with stirring at ambient temperature for 15 min. Progress of the reaction was monitored by thin layer chromatography. Upon completion of the reaction saturated sodium bicarbonate solution was added and a white solid formed. It was filtered off and dried (Yield 0.58 g, 87.8%). This solid was recrystallized in ethanol to yield block-like colourless crystals of the title compound.

### 3. Refinement

H atoms were positioned geometrically and allowed to ride on their parent atoms: C—H = 0.93–0.98 Å with *U*_iso_(H) =1.5*U*_eq_(C-methyl) and = 1.2*U*_eq_(C) for other H atoms.

### Crystal data

**Table:**

C_27_H_29_N_3_O_2_	*F*(000) = 912
*M**_r_* = 427.53	*D*_x_ = 1.197 Mg m^−^^3^
Orthorhombic, *P**n**a*2_1_	Mo *K*α radiation, λ = 0.71073 Å
Hall symbol: P 2c -2n	Cell parameters from 5117 reflections
*a* = 12.7717 (6) Å	θ = 2.0–28.3°
*b* = 16.2765 (7) Å	µ = 0.08 mm^−^^1^
*c* = 11.4109 (9) Å	*T* = 293 K
*V* = 2372.1 (2) Å^3^	Block, colourless
*Z* = 4	0.20 × 0.20 × 0.20 mm

### Data collection

**Table:**

Bruker SMART APEXII CCD diffractometer	5117 independent reflections
Radiation source: fine-focus sealed tube	2975 reflections with *I* > 2σ(*I*)
Graphite monochromator	*R*_int_ = 0.045
ω amd φ scans	θ_max_ = 28.3°, θ_min_ = 2.0°
Absorption correction: multi-scan (*SADABS*; Bruker, 2008)	*h* = −16→14
*T*_min_ = 0.491, *T*_max_ = 0.746	*k* = −21→16
11654 measured reflections	*l* = −12→15

### Refinement

**Table:**

Refinement on *F*^2^	Primary atom site location: structure-invariant direct methods
Least-squares matrix: full	Secondary atom site location: difference Fourier map
*R*[*F*^2^ > 2σ(*F*^2^)] = 0.047	Hydrogen site location: inferred from neighbouring sites
*wR*(*F*^2^) = 0.132	H-atom parameters constrained
*S* = 1.03	*w* = 1/[σ^2^(*F*_o_^2^) + (0.0556*P*)^2^ + 0.1257*P*] where *P* = (*F*_o_^2^ + 2*F*_c_^2^)/3
5117 reflections	(Δ/σ)_max_ < 0.001
292 parameters	Δρ_max_ = 0.15 e Å^−^^3^
1 restraint	Δρ_min_ = −0.14 e Å^−^^3^

### Special details

**Table:**

Geometry. All e.s.d.'s (except the e.s.d. in the dihedral angle between two l.s. planes) are estimated using the full covariance matrix. The cell e.s.d.'s are taken into account individually in the estimation of e.s.d.'s in distances, angles and torsion angles; correlations between e.s.d.'s in cell parameters are only used when they are defined by crystal symmetry. An approximate (isotropic) treatment of cell e.s.d.'s is used for estimating e.s.d.'s involving l.s. planes.
Refinement. Refinement of *F*^2^ against ALL reflections. The weighted *R*-factor *wR* and goodness of fit *S* are based on *F*^2^, conventional *R*-factors *R* are based on *F*, with *F* set to zero for negative *F*^2^. The threshold expression of *F*^2^ > σ(*F*^2^) is used only for calculating *R*-factors(gt) *etc*. and is not relevant to the choice of reflections for refinement. *R*-factors based on *F*^2^ are statistically about twice as large as those based on *F*, and *R*- factors based on ALL data will be even larger.

### Fractional atomic coordinates and isotropic or equivalent isotropic displacement parameters (Å^2^)

**Table:**

	*x*	*y*	*z*	*U*_iso_*/*U*_eq_	
C1	0.20093 (18)	0.54079 (15)	0.4090 (2)	0.0521 (6)	
H1	0.1920	0.5854	0.3523	0.063*	
C2	0.29009 (17)	0.48375 (15)	0.3647 (2)	0.0540 (6)	
H2	0.2969	0.4405	0.4240	0.065*	
C3	0.39055 (18)	0.53079 (15)	0.3689 (2)	0.0512 (6)	
C4	0.41492 (19)	0.56519 (17)	0.4867 (2)	0.0563 (6)	
H4A	0.4796	0.5963	0.4832	0.068*	
H4B	0.4241	0.5208	0.5425	0.068*	
C5	0.32572 (18)	0.62083 (15)	0.5264 (2)	0.0509 (6)	
H5	0.3216	0.6677	0.4727	0.061*	
C6	0.09879 (17)	0.49393 (16)	0.4170 (2)	0.0547 (6)	
C7	0.0836 (2)	0.43621 (18)	0.5037 (2)	0.0648 (7)	
H7	0.1361	0.4267	0.5585	0.078*	
C8	−0.0087 (2)	0.39242 (19)	0.5100 (3)	0.0775 (9)	
H8	−0.0178	0.3534	0.5688	0.093*	
C9	−0.0869 (2)	0.4060 (2)	0.4306 (4)	0.0844 (10)	
H9	−0.1492	0.3765	0.4355	0.101*	
C10	−0.0733 (2)	0.4626 (2)	0.3444 (3)	0.0864 (10)	
H10	−0.1259	0.4713	0.2894	0.104*	
C11	0.0187 (2)	0.5076 (2)	0.3384 (3)	0.0729 (8)	
H11	0.0265	0.5475	0.2807	0.087*	
C12	0.2629 (2)	0.43909 (18)	0.2496 (3)	0.0656 (7)	
H12	0.1952	0.4125	0.2628	0.079*	
C13	0.3406 (2)	0.3698 (2)	0.2243 (4)	0.0901 (11)	
H13A	0.3168	0.3386	0.1580	0.135*	
H13B	0.3456	0.3345	0.2914	0.135*	
H13C	0.4082	0.3928	0.2075	0.135*	
C14	0.2487 (2)	0.4935 (2)	0.1429 (3)	0.0937 (10)	
H14A	0.3158	0.5127	0.1167	0.141*	
H14B	0.2054	0.5396	0.1630	0.141*	
H14C	0.2159	0.4625	0.0814	0.141*	
C15	0.5761 (2)	0.62544 (18)	0.2066 (3)	0.0616 (7)	
C16	0.6794 (2)	0.66287 (17)	0.2345 (3)	0.0655 (8)	
C17	0.7231 (3)	0.7185 (2)	0.1568 (4)	0.0922 (10)	
H17	0.6883	0.7329	0.0883	0.111*	
C18	0.8190 (4)	0.7520 (2)	0.1830 (4)	0.1076 (14)	
H18	0.8497	0.7906	0.1338	0.129*	
C19	0.8680 (3)	0.7271 (3)	0.2831 (5)	0.1080 (14)	
H19	0.9336	0.7492	0.2989	0.130*	
N3	0.8297 (2)	0.67423 (19)	0.3587 (3)	0.1028 (10)	
C21	0.7358 (3)	0.64310 (19)	0.3324 (3)	0.0793 (9)	
H21	0.7067	0.6054	0.3843	0.095*	
C22	0.35138 (17)	0.65246 (16)	0.6471 (2)	0.0532 (6)	
C23	0.3903 (2)	0.73080 (17)	0.6616 (3)	0.0643 (7)	
H23	0.3944	0.7659	0.5975	0.077*	
C24	0.4232 (2)	0.7578 (2)	0.7699 (3)	0.0749 (8)	
H24	0.4491	0.8109	0.7781	0.090*	
C25	0.4180 (2)	0.7075 (2)	0.8649 (3)	0.0792 (9)	
H25	0.4408	0.7259	0.9377	0.095*	
C26	0.3788 (2)	0.6292 (2)	0.8524 (3)	0.0748 (8)	
H26	0.3743	0.5948	0.9173	0.090*	
C27	0.34641 (19)	0.60174 (18)	0.7443 (2)	0.0615 (7)	
H27	0.3209	0.5485	0.7364	0.074*	
C28	0.1417 (2)	0.63578 (19)	0.5558 (3)	0.0726 (8)	
H28A	0.1567	0.6592	0.6311	0.109*	
H28B	0.0758	0.6075	0.5588	0.109*	
H28C	0.1384	0.6787	0.4982	0.109*	
N1	0.22489 (14)	0.57741 (12)	0.52408 (17)	0.0523 (5)	
N2	0.44194 (16)	0.54136 (15)	0.27484 (19)	0.0606 (6)	
O1	0.53751 (13)	0.58708 (12)	0.29990 (16)	0.0637 (5)	
O2	0.53326 (18)	0.63179 (16)	0.1144 (2)	0.0924 (7)	

### Atomic displacement parameters (Å^2^)

**Table:**

	*U*^11^	*U*^22^	*U*^33^	*U*^12^	*U*^13^	*U*^23^
C1	0.0479 (13)	0.0507 (15)	0.0576 (15)	0.0025 (11)	−0.0069 (11)	−0.0015 (11)
C2	0.0462 (13)	0.0553 (15)	0.0607 (15)	0.0013 (11)	0.0002 (11)	−0.0026 (13)
C3	0.0453 (12)	0.0525 (15)	0.0556 (14)	0.0039 (10)	−0.0006 (11)	−0.0039 (12)
C4	0.0454 (13)	0.0680 (18)	0.0554 (14)	−0.0058 (12)	−0.0026 (11)	−0.0015 (12)
C5	0.0535 (14)	0.0458 (14)	0.0536 (13)	−0.0060 (11)	−0.0033 (11)	0.0019 (11)
C6	0.0440 (14)	0.0579 (16)	0.0623 (15)	0.0045 (11)	−0.0007 (11)	−0.0118 (13)
C7	0.0552 (15)	0.073 (2)	0.0660 (18)	−0.0035 (14)	0.0046 (12)	−0.0131 (15)
C8	0.0678 (19)	0.075 (2)	0.090 (2)	−0.0116 (16)	0.0204 (17)	−0.0194 (17)
C9	0.0539 (18)	0.084 (3)	0.115 (3)	−0.0101 (16)	0.0090 (19)	−0.034 (2)
C10	0.0494 (16)	0.096 (3)	0.113 (3)	0.0037 (17)	−0.0174 (17)	−0.031 (2)
C11	0.0577 (16)	0.075 (2)	0.086 (2)	0.0084 (15)	−0.0131 (14)	−0.0123 (16)
C12	0.0518 (15)	0.0688 (19)	0.0762 (18)	−0.0028 (13)	−0.0025 (13)	−0.0221 (15)
C13	0.069 (2)	0.081 (2)	0.120 (3)	0.0027 (16)	0.0055 (18)	−0.042 (2)
C14	0.088 (2)	0.125 (3)	0.0678 (19)	0.015 (2)	−0.0179 (16)	−0.019 (2)
C15	0.0657 (16)	0.0581 (18)	0.0611 (17)	0.0092 (14)	0.0157 (14)	0.0019 (13)
C16	0.0666 (17)	0.0517 (17)	0.0781 (19)	−0.0034 (13)	0.0257 (16)	−0.0086 (14)
C17	0.098 (3)	0.081 (2)	0.097 (2)	−0.005 (2)	0.035 (2)	0.0062 (19)
C18	0.119 (3)	0.086 (3)	0.118 (4)	−0.035 (2)	0.053 (3)	−0.003 (2)
C19	0.096 (3)	0.093 (3)	0.135 (4)	−0.039 (2)	0.039 (3)	−0.032 (3)
N3	0.0872 (19)	0.095 (2)	0.126 (3)	−0.0257 (17)	−0.0013 (19)	−0.019 (2)
C21	0.080 (2)	0.071 (2)	0.087 (2)	−0.0186 (17)	0.0047 (17)	−0.0029 (17)
C22	0.0451 (13)	0.0557 (16)	0.0589 (15)	0.0005 (11)	−0.0003 (11)	−0.0035 (12)
C23	0.0682 (16)	0.0553 (17)	0.0694 (17)	−0.0019 (14)	−0.0091 (13)	−0.0013 (13)
C24	0.0781 (19)	0.064 (2)	0.083 (2)	−0.0002 (15)	−0.0138 (15)	−0.0186 (17)
C25	0.083 (2)	0.092 (3)	0.0635 (18)	0.0030 (17)	−0.0075 (16)	−0.0227 (18)
C26	0.0719 (17)	0.097 (3)	0.0553 (16)	0.0007 (16)	0.0053 (14)	0.0051 (16)
C27	0.0590 (16)	0.0676 (19)	0.0580 (16)	−0.0072 (13)	0.0066 (12)	0.0030 (14)
C28	0.0561 (15)	0.0680 (19)	0.094 (2)	0.0086 (14)	−0.0002 (14)	−0.0209 (15)
N1	0.0442 (10)	0.0519 (13)	0.0608 (12)	0.0003 (9)	−0.0013 (9)	−0.0066 (10)
N2	0.0501 (12)	0.0657 (15)	0.0660 (14)	−0.0036 (10)	−0.0007 (10)	−0.0088 (11)
O1	0.0567 (10)	0.0744 (13)	0.0601 (11)	−0.0105 (9)	0.0055 (8)	−0.0032 (9)
O2	0.0921 (15)	0.1099 (19)	0.0752 (16)	−0.0005 (13)	−0.0031 (12)	0.0219 (13)

### Geometric parameters (Å, º)

**Table:**

C1—N1	1.474 (3)	C14—H14A	0.9600
C1—C6	1.514 (3)	C14—H14B	0.9600
C1—C2	1.554 (3)	C14—H14C	0.9600
C1—H1	0.9800	C15—O2	1.189 (3)
C2—C3	1.495 (3)	C15—O1	1.329 (3)
C2—C12	1.541 (4)	C15—C16	1.488 (4)
C2—H2	0.9800	C16—C21	1.367 (4)
C3—N2	1.270 (3)	C16—C17	1.385 (4)
C3—C4	1.489 (3)	C17—C18	1.372 (5)
C4—C5	1.524 (4)	C17—H17	0.9300
C4—H4A	0.9700	C18—C19	1.363 (6)
C4—H4B	0.9700	C18—H18	0.9300
C5—N1	1.469 (3)	C19—N3	1.313 (5)
C5—C22	1.507 (3)	C19—H19	0.9300
C5—H5	0.9800	N3—C21	1.336 (4)
C6—C11	1.378 (4)	C21—H21	0.9300
C6—C7	1.378 (4)	C22—C23	1.378 (4)
C7—C8	1.379 (4)	C22—C27	1.383 (4)
C7—H7	0.9300	C23—C24	1.377 (4)
C8—C9	1.366 (5)	C23—H23	0.9300
C8—H8	0.9300	C24—C25	1.360 (4)
C9—C10	1.360 (5)	C24—H24	0.9300
C9—H9	0.9300	C25—C26	1.377 (4)
C10—C11	1.387 (4)	C25—H25	0.9300
C10—H10	0.9300	C26—C27	1.377 (4)
C11—H11	0.9300	C26—H26	0.9300
C12—C14	1.516 (5)	C27—H27	0.9300
C12—C13	1.529 (4)	C28—N1	1.471 (3)
C12—H12	0.9800	C28—H28A	0.9600
C13—H13A	0.9600	C28—H28B	0.9600
C13—H13B	0.9600	C28—H28C	0.9600
C13—H13C	0.9600	N2—O1	1.458 (3)
			
N1—C1—C6	109.24 (19)	H13B—C13—H13C	109.5
N1—C1—C2	112.30 (18)	C12—C14—H14A	109.5
C6—C1—C2	110.48 (19)	C12—C14—H14B	109.5
N1—C1—H1	108.2	H14A—C14—H14B	109.5
C6—C1—H1	108.2	C12—C14—H14C	109.5
C2—C1—H1	108.2	H14A—C14—H14C	109.5
C3—C2—C12	117.5 (2)	H14B—C14—H14C	109.5
C3—C2—C1	108.21 (19)	O2—C15—O1	125.3 (3)
C12—C2—C1	113.24 (19)	O2—C15—C16	124.2 (3)
C3—C2—H2	105.6	O1—C15—C16	110.5 (3)
C12—C2—H2	105.6	C21—C16—C17	117.7 (3)
C1—C2—H2	105.6	C21—C16—C15	123.1 (3)
N2—C3—C4	127.2 (2)	C17—C16—C15	119.2 (3)
N2—C3—C2	119.1 (2)	C18—C17—C16	118.7 (4)
C4—C3—C2	113.6 (2)	C18—C17—H17	120.7
C3—C4—C5	109.6 (2)	C16—C17—H17	120.7
C3—C4—H4A	109.7	C19—C18—C17	118.3 (4)
C5—C4—H4A	109.7	C19—C18—H18	120.9
C3—C4—H4B	109.7	C17—C18—H18	120.9
C5—C4—H4B	109.7	N3—C19—C18	125.0 (4)
H4A—C4—H4B	108.2	N3—C19—H19	117.5
N1—C5—C22	111.80 (19)	C18—C19—H19	117.5
N1—C5—C4	111.34 (19)	C19—N3—C21	115.8 (4)
C22—C5—C4	108.20 (19)	N3—C21—C16	124.5 (3)
N1—C5—H5	108.5	N3—C21—H21	117.7
C22—C5—H5	108.5	C16—C21—H21	117.7
C4—C5—H5	108.5	C23—C22—C27	118.2 (2)
C11—C6—C7	118.2 (2)	C23—C22—C5	120.3 (2)
C11—C6—C1	121.3 (3)	C27—C22—C5	121.2 (2)
C7—C6—C1	120.5 (2)	C24—C23—C22	120.8 (3)
C6—C7—C8	120.7 (3)	C24—C23—H23	119.6
C6—C7—H7	119.7	C22—C23—H23	119.6
C8—C7—H7	119.7	C25—C24—C23	120.6 (3)
C9—C8—C7	120.4 (3)	C25—C24—H24	119.7
C9—C8—H8	119.8	C23—C24—H24	119.7
C7—C8—H8	119.8	C24—C25—C26	119.5 (3)
C10—C9—C8	119.8 (3)	C24—C25—H25	120.2
C10—C9—H9	120.1	C26—C25—H25	120.2
C8—C9—H9	120.1	C27—C26—C25	120.2 (3)
C9—C10—C11	120.1 (3)	C27—C26—H26	119.9
C9—C10—H10	119.9	C25—C26—H26	119.9
C11—C10—H10	119.9	C26—C27—C22	120.7 (3)
C6—C11—C10	120.8 (3)	C26—C27—H27	119.6
C6—C11—H11	119.6	C22—C27—H27	119.6
C10—C11—H11	119.6	N1—C28—H28A	109.5
C14—C12—C13	110.9 (3)	N1—C28—H28B	109.5
C14—C12—C2	115.9 (2)	H28A—C28—H28B	109.5
C13—C12—C2	111.3 (2)	N1—C28—H28C	109.5
C14—C12—H12	106.0	H28A—C28—H28C	109.5
C13—C12—H12	106.0	H28B—C28—H28C	109.5
C2—C12—H12	106.0	C5—N1—C28	108.55 (19)
C12—C13—H13A	109.5	C5—N1—C1	113.11 (18)
C12—C13—H13B	109.5	C28—N1—C1	109.30 (19)
H13A—C13—H13B	109.5	C3—N2—O1	109.64 (19)
C12—C13—H13C	109.5	C15—O1—N2	113.1 (2)
H13A—C13—H13C	109.5		
			
N1—C1—C2—C3	−52.7 (3)	C15—C16—C17—C18	−179.3 (3)
C6—C1—C2—C3	−174.9 (2)	C16—C17—C18—C19	1.8 (5)
N1—C1—C2—C12	175.2 (2)	C17—C18—C19—N3	−1.5 (6)
C6—C1—C2—C12	52.9 (3)	C18—C19—N3—C21	0.7 (6)
C12—C2—C3—N2	9.3 (3)	C19—N3—C21—C16	−0.2 (5)
C1—C2—C3—N2	−120.5 (2)	C17—C16—C21—N3	0.5 (5)
C12—C2—C3—C4	−174.6 (2)	C15—C16—C21—N3	178.5 (3)
C1—C2—C3—C4	55.7 (3)	N1—C5—C22—C23	−135.9 (2)
N2—C3—C4—C5	118.2 (3)	C4—C5—C22—C23	101.1 (3)
C2—C3—C4—C5	−57.6 (3)	N1—C5—C22—C27	50.4 (3)
C3—C4—C5—N1	55.1 (3)	C4—C5—C22—C27	−72.5 (3)
C3—C4—C5—C22	178.3 (2)	C27—C22—C23—C24	−0.1 (4)
N1—C1—C6—C11	128.0 (2)	C5—C22—C23—C24	−173.9 (2)
C2—C1—C6—C11	−108.0 (3)	C22—C23—C24—C25	0.1 (5)
N1—C1—C6—C7	−51.9 (3)	C23—C24—C25—C26	−0.5 (5)
C2—C1—C6—C7	72.1 (3)	C24—C25—C26—C27	0.8 (5)
C11—C6—C7—C8	1.2 (4)	C25—C26—C27—C22	−0.8 (4)
C1—C6—C7—C8	−178.9 (2)	C23—C22—C27—C26	0.4 (4)
C6—C7—C8—C9	−0.4 (4)	C5—C22—C27—C26	174.2 (2)
C7—C8—C9—C10	0.4 (5)	C22—C5—N1—C28	62.5 (3)
C8—C9—C10—C11	−1.2 (5)	C4—C5—N1—C28	−176.4 (2)
C7—C6—C11—C10	−2.0 (4)	C22—C5—N1—C1	−176.0 (2)
C1—C6—C11—C10	178.1 (3)	C4—C5—N1—C1	−54.9 (2)
C9—C10—C11—C6	2.0 (4)	C6—C1—N1—C5	177.12 (19)
C3—C2—C12—C14	−62.2 (3)	C2—C1—N1—C5	54.2 (3)
C1—C2—C12—C14	65.2 (3)	C6—C1—N1—C28	−61.8 (3)
C3—C2—C12—C13	65.7 (3)	C2—C1—N1—C28	175.2 (2)
C1—C2—C12—C13	−167.0 (2)	C4—C3—N2—O1	5.6 (3)
O2—C15—C16—C21	−168.0 (3)	C2—C3—N2—O1	−178.9 (2)
O1—C15—C16—C21	14.4 (4)	O2—C15—O1—N2	8.8 (4)
O2—C15—C16—C17	10.0 (4)	C16—C15—O1—N2	−173.59 (19)
O1—C15—C16—C17	−167.7 (3)	C3—N2—O1—C15	−157.5 (2)
C21—C16—C17—C18	−1.3 (5)		

### Hydrogen-bond geometry (Å, º)

Cg1 and Cg2 are the centroids of rings C6–C11 and C22–C27, respectively.

**Table:**

*D*—H···*A*	*D*—H	H···*A*	*D*···*A*	*D*—H···*A*
C24—H24···*Cg*1^i^	0.93	2.87	3.700 (4)	150
C13—H13*C*···*Cg*2^ii^	0.96	2.95	3.620 (3)	128

Symmetry codes: (i) −*x*+1/2, *y*+1/2, *z*+1/2; (ii) −*x*+1, −*y*+1, *z*−1/2.
